# LASP2 suppresses colorectal cancer progression through JNK/p38 MAPK pathway meditated epithelial-mesenchymal transition

**DOI:** 10.1186/s12964-017-0179-9

**Published:** 2017-06-12

**Authors:** Bin Wang, Lanzhi Zhang, Liying Zhao, Rui Zhou, Yanqing Ding, Guoxin Li, Liang Zhao

**Affiliations:** 1 Department of Pathology, Nanfang Hospital, Southern Medical University, Guangzhou, China; 2 Department of General Surgery, Nanfang Hospital, Southern Medical University, Guangdong provincial Engineering Technology Research Center of Minimally Invasive Surgery, Guangzhou, China; 3 Department of Pathology, School of Basic Medical Sciences, Southern Medical University, Guangzhou, China

**Keywords:** LIM and SH3 Protein 2, Colorectal cancer, Tumor metastasis, Epithelial–mesenchymal transition, LIM and SH3 Protein 1

## Abstract

**Background:**

LASP2 (LIM and SH3 Protein 2) is a small focal adhesion protein belongs to nebulin protein family. As the newest member of nebulette family, the function of LASP2 remains to be identified.

**Methods:**

The relationship between LASP2 expression and clinical characteristics of CRC was analyzed in 89 paraffin-embedded archived CRC specimens by immunohistochemistry (IHC). The effects of LASP2 on cell growth and migration were examined in vitro, using CCK-8 and transwell assays. Western blotting was performed to examine the impact of LASP2 on the SAPK/JNK and MAPK signaling pathways.

**Results:**

In the present study, we observed a decreased LASP2 expression in clinical colorectal cancer samples compared with paired normal tissues. A negative correlation was also found between LASP2 and poor prognosis of CRC patients. Gain- and loss-of-function approaches revealed that LASP2 plays inhibitory effects on the growth and migration of human CRC cells in vitro. Western-blot results showed that LASP2 could attenuate epithelial-mesenchymal transition (EMT) to accomplish its suppression on CRC aggression. In LASP2 knocked down CRC cells, EMT was inhibited along with the inactivation of JNK/p38 MAPK pathway. Consistently, treatment of JNK inhibitor (JNK inhibitor II) together with p38 inhibitor (SB203580) could resume the process of EMT. Interestingly, we found a negative relationship between LASP2 and LASP1 expression in both CRC cell lines and tumors tissues, which suggests their converse function in CRC progression.

**Conclusions:**

All the findings indicated that LASP2 may play a significant role in suppressing CRC progression and provided a novel biomarker for CRC therapy.

**Electronic supplementary material:**

The online version of this article (doi:10.1186/s12964-017-0179-9) contains supplementary material, which is available to authorized users.

## Background

Colorectal cancer (CRC) is one of the most common cancers and a crucial cause of cancer-related death worldwide [1]. The incidence of CRC has been fleetly increasing over the past decades due to the so-called western lifestyle [2, 3]. Although astounding improvements has been made in diagnosis and therapy, the overall survival of CRC patients remains poor due to the late diagnosis and metastasis [4, 5]. Thus, it’s essential to identify molecular mechanisms responsible for CRC metastasis and seek new ways to facilitate early diagnosis.

LASP2 (LIM and SH3 Protein 2), an isoform of nebulette, belongs to nebulin protein family of actin binding protein [6]. Incipient studies of LASP2 were mainly focused on its impact on cardiac diseases since nebulette was first identified only expressed in myocardium [7, 8]. However, emphasis switched to its influence on cytoskeleton assembling and cell migration since different expression pattern of LASP2 and nebulette was detected [9, 10]. Unlike nebulette, LASP2 was detected not only in cardiac muscle but also many other organs including brain, lung and kidney. Interestingly, LASP2 was more closely to LASP1 although they are splice-variants of nebulette sharing two transcriptional genes [11]. LASP1 was demonstrated involving in many cellular processes such as cell proliferation, progression and migration of several carcinomas. Our work had proved that LASP1 promotes CRC proliferation and metastasis, maybe suggesting its correlations in the CRC progression [12]. LASP1, a downstream factor of TGF-beta, induced epithelial-mesenchymal transition and contributed CRC metastasis [13]. LASP2, as a member of the same protein family, is more likely to participate in these processes the same as LASP1 for both containing LIM and SH3 domain [14].

In this research, we investigated the expression of LASP2 in CRC tissues and cell lines for the first time, which to some extent elucidated the function of LASP2 in CRC. Furthermore, suppressive effects of LASP2 were found when compared the survival curve of CRC patients with high and low expression of LASP2. We achieved a comprehensive understanding of LASP2 in the progression of CRC and propose it as a brand new marker for CRC staging and prognosis.

## Methods

### Cell lines and cell culture

CRC cell lines HCT116, HT29, LS174T, RKO, SW480, SW620 and LOVO were accessed from the Cell Bank of Chinese Academy of Sciences (Shanghai, China) and maintained as described previously [12]. Additionally, a human CRC cell subline having unique liver metastatic potential, identified as SW480/M5, was established in our laboratory [15] and applied in this research. All the cells were cultured in RPMI 1640 (Hyclone) supplemented with 10% fetal bovine serum (FBS) (Gibco-BRL, Invitrogen) at a humidity of 5% CO_2_ at 37 °C.

For inhibitor treatment, both JNK inhibitor II (Millipore) [16] and p38 inhibitor SB203580 (Millipore) [17] were first dissolved in DMSO and then added to the cultured cells at a desired concentration 20 μM for JNK inhibitor II and 10 μM for SB203580 every 2 days, respectively.

### RNA extraction, RT-PCR and real-time PCR

Total RNAs were isolated from CRC cell lines with Trizol reagent (Invitrogen Life Technologies) according to manufacturer’s protocol. To quantitate the expression of lasp2, total RNA was polyadenylated and reverse transcription was performed using 2μg of RNA of each sample by SuperScript II RT kit (Invitrogen Life Technologies). GAPDH was chosen as an internal quantitative control. All the primers were purchased from Invitrogen and the primer sequences of lasp2 were as follows: sense primer 5′-CATTCCCAAGGCTATGGCTA-3′ and anti-sense primer 5′-ATCGTACATGGCTCGGTAGG-3′. The primer sequences for GAPDH were as follows: sense primer 5′-CCACCCATGGCAAATTCCATGGCA-3′ and anti-sense primer 5′-TCTAGACGGCAGGTCAGGTCCAC-3′. Expression data analyzed by the 2^-△△CT^ method described previously [18].

### Western blotting

Immunoblot analysis was performed to assess protein expression of cell lysates (20-60 μg) in RIPA buffer in the presence of mouse antibodies to β-tubulin (1:1000; Santa Cruz); rabbit antibody to LASP2 (1:500; Abcam); rabbit antibodies to p-SAPK/JNK, SAPK/JNK, p-p38, p38, p-p44/42, p44/42 (1:500; CST) and rabbit antibody to ZO-1, E-cadherin, β-catenin, N-cadherin, Vimentin (1:1000; CST).

### Immunohistochemistry

IHC was used to detect the expression of proteins in 89 human colorectal cancer tissues as described previously [19]. The slides were incubated overnight with primary antibodies against LASP2 (1:500) and LASP1 (1:500), at 4 °C. Mayer’s hematoxylin was applied for nuclear counterstaining. In our research, these sections were reviewed by three blind-folded pathologists, and the intensity of staining of malignant cells was scored as below to analyze the levels of protein expression: 0 (no staining), 1 (weak staining, faint yellow), 2 (moderate staining, light brown), and 3 (strong staining, brown). A score of intensity >2 was classified as high expression, whereas <2 was considered as low expression. The discrepancies (<5%) were settled by reevaluation simultaneously.

### Vectors construction and siRNA transfection

Full-length human lasp2 cDNA was amplified and subcloned into pENTER to construct LASP2 overexpression vector by standard PCR techniques (Shanghai GenePharma Co., Shanghai, China). SiRNA sequences 5′-GUCCUAUGCUAAACCAUGUTT-3′ and 5′-ACAUGGUUUAGCAUAGGACTT-3′ were used to knockdown LASP2. Transfection was performed as previously described [20].

### CCK-8 assay

CRC cells were planted to 96-well plates at a concentration of 1 × 10^3^ cells per cell. Cell viability of control, LASP2 overexpressed and LASP2 knock-down cells were detected using Cell Counting Kit-8 assay. Briefly, 10 μl Cell Counting Kit-8 (Dojindo) was added to each well for 2 h and the absorbance value (OD) was measured at 490 nm using a microplate reader. All the experiments were repeated for at least three times.

### Transwell assay

CRC cells (1 × 10^5^ cells/100 ml) were added to the upper chamber of Transwell inserts (8 mm pore size) with serum-free medium (Corning Star). Medium with 10% FBS was infused to the lower chamber as a chemoattractant. Cells added to the upper chamber were allowed to migrate through the porous membrane for 24 h or 48 h at 37 °C. The cells stocked to the lower surface of the membrane after migrating through the porous membrane were fixed with methanol for 20 min and stained with Giemsa solution for 15 min for visualization. Five randomly selected fields of the cells were counted under a microscope(original magnification, ×200) for quantification.

### Statistical analysis

Data were analyzed using SPSS 19.0. Statistical significance of difference between groups was determined by a two-tailed paired Student’s *t* test. Chi-square test was performed to examine the relationships between LASP2 expression and clinicopathologic characteristics. Kaplan-Meier plots were performed to investigate the prognostic relevance of LASP2 in univariate analysis. Cox regression model was performed to assess the statistical significance of various survival-related variables in multivariate analysis. *P* < 0.05 was acknowledged as statistically significant.

## Results

### LASP2 is downregulated in colorectal cancer tissues

**Fig. 1:**
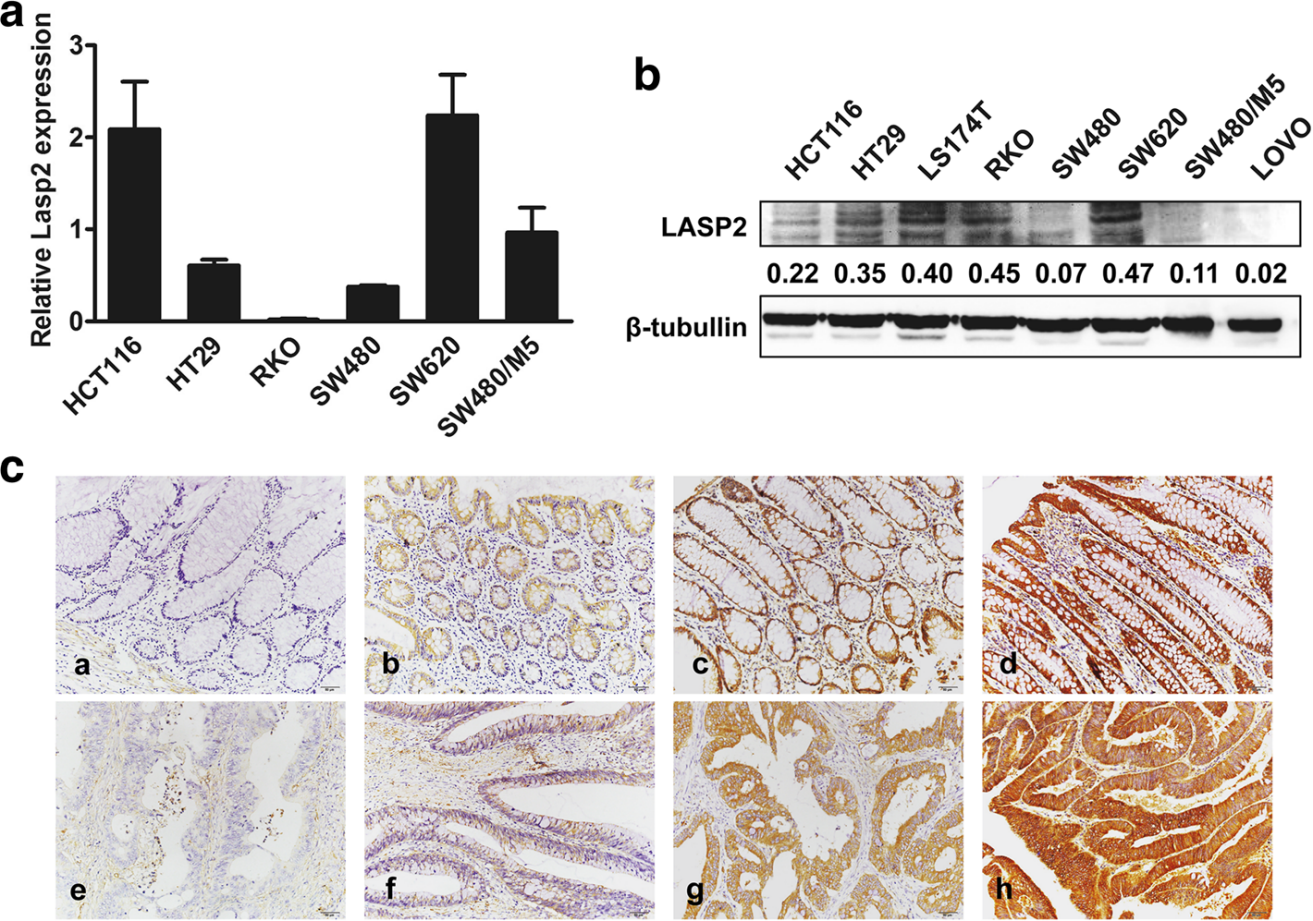
LASP2 is downregulated in colorectal cancer tissues. (**a**) LASP2 mRNA expression in CRC cell lines by real-time PCR. Expression levels were determined by the normalization of GAPDH. *Error bars* represent mean SD averaged from 3 independent experiments. (**b**) LASP2 expression in CRC cell lines by western blot. Immunosignal represent the relative amount of protein expression and was quantified by greyscale scanning software, and relative protein abundance was normalized by β-tubulin. (**c**) LASP2 expression in normal and CRC tissues by IHC from paraffin blocks. Representatives of LASP1 staining intensity: (a) negative in adjacent tissue, (b) weak in adjacent tissue, (c) moderate in adjacent tissue, (d) strong in adjacent tissue, (e) negative in tumor tissue, (f) weak in tumor tissue, (g) moderate in tumor tissue and (h) strong in tumor tissue

The results of qPCR and immunoblotting assays showed that LASP2 is differently expressed in various CRC cell lines. High transcriptional level of LASP2 expression in a certain cell line was not totally consistent with that of high translational level. However, LASP2 always highly expressed in the metastatic CRC cell line SW620 and relatively lowly expressed in the primary tumor cell line SW480 (Fig. 1a and b). Hence, we select those the two cell lines to launch the following functional experiments.

Further, the expression and subcellular localization of LASP2 were determined by IHC analysis in 72 and 89 paraffin-embedded, archival normal colorectal mucosa and CRC tissues, respectively. The intensity of tissue staining was classified to better discriminate no staining, weak, moderate, and strong staining in both the normal and tumor tissues (Fig. 1c). LASP2 was overexpressed in 68.0% (49/72) of normal colorectal samples. Compared with these normal tissues, a relatively high expression level of LASP2 was only observed in 50.6% (45/89) of CRC samples (*P* = 0.025) (Additional file 1: Table S1).

### LASP2 was negatively correlated with progression and poor survival in CRC

**Fig. 2:**
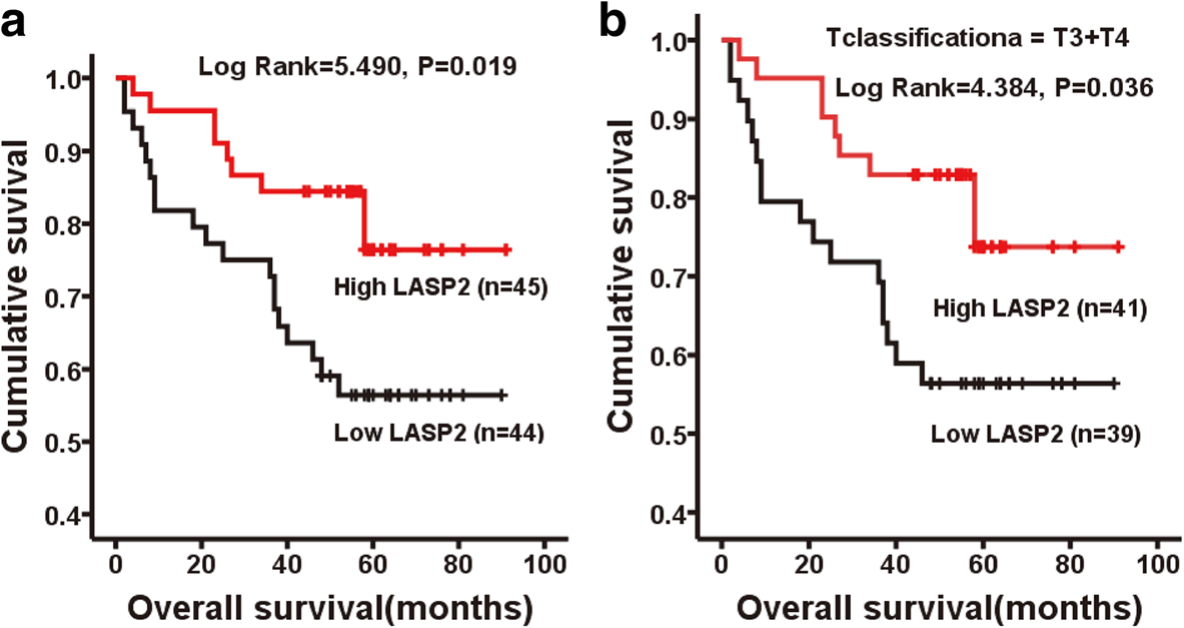
LASP2 was negatively associated with progression and poor survival in CRC. Kaplan-Meier survival analysis in patients with CRC. **a** Overall survival of all patients. (*n* = 89, log rank = 5.490, *P* = 0.019). **b** Overall survival of patients in T3 and T4 stage (*n* = 80, log rank = 4.384, *P* = 0.036)

Chi-square test revealed that the protein levels of LASP2 significantly correlated with differentiation (*P* = 0.023), N classification(*P* = 0.026) and AJCC stage (*P* = 0.015) (Additional file 1: Table S1). Kaplan–Meier survival analysis demonstrated that the CRC patients with high expression level of LASP2 had a better prognosis not only in T3 + T4 stage but also all stages (Fig. 2). Although Cox proportional hazards model of multivariate analysis indicated that LASP2 expression level is not an independent prognostic factors for prognosis of CRC patients, univariate analysis revealed it do correlate with the overall survival rate (Additional file 1: Table S2).

### LASP2 suppresses human CRC cell growth and migration

**Fig. 3:**
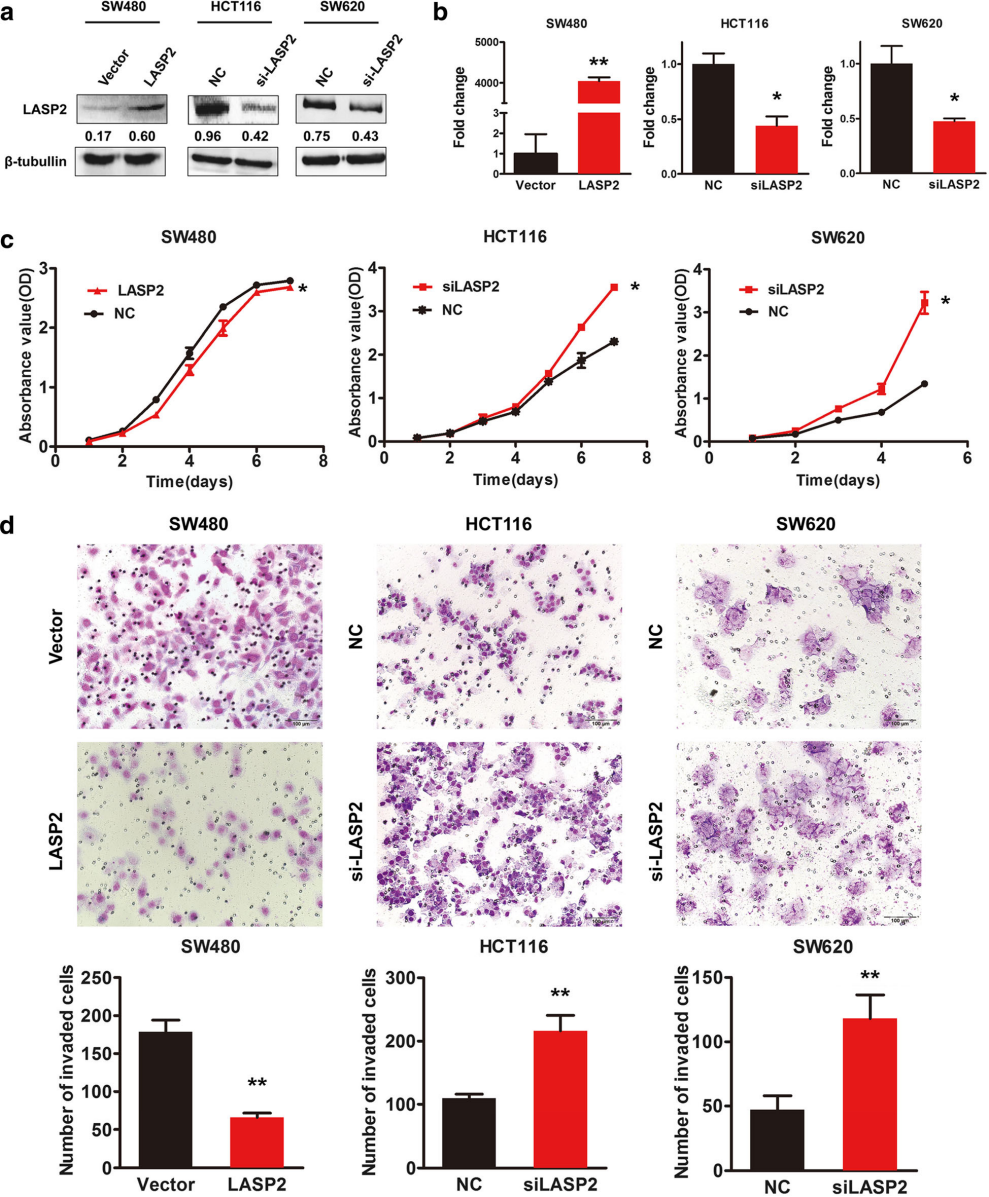
LASP2 suppresses human CRC cell growth and migration. Transfection of LASP2 vector and siRNA was detected by western blot (**a**) and real time RT-PCR (**b**). Function of exogenous LASP2 overexpression and knockdown was investigated by CCK-8 assay (**c**) and transwell assay (**d**). Representative figures were shown. *Error bars* represent mean SD averaged from 3 independent experiments. (*) indicates *P* < 0.05. (**) indicates *P* < 0.01

To investigate the potential effect of LASP2 on the proliferation and migration of human CRC cells, gain- and loss-of-function assays were performed in vitro. We transfected LASP2 vector to SW480 and siRNA to HCT116 together with SW620 cells (Fig. 3a and b). CCK-8 assay showed that overexpressed LASP2 in SW480 significantly decreased the proliferation compared with the control cells. Conversely, cell proliferation was significantly increased in the LASP2 knockdown HCT116 and SW620 cells (Fig. 3c). Transwell assay revealed that exogenous introduction of LASP2 tends to decrease cell migration in SW480 cells. On the contrary, depletion of endogenous LASP2 expression mediated by siRNA could enhance the migratory ability in HCT116 and SW620 cells (Fig. 3d).

### JNK/p38 MAPK inactivation is required for LASP2-medatied cell behaviors in CRC cells

**Fig. 4:**
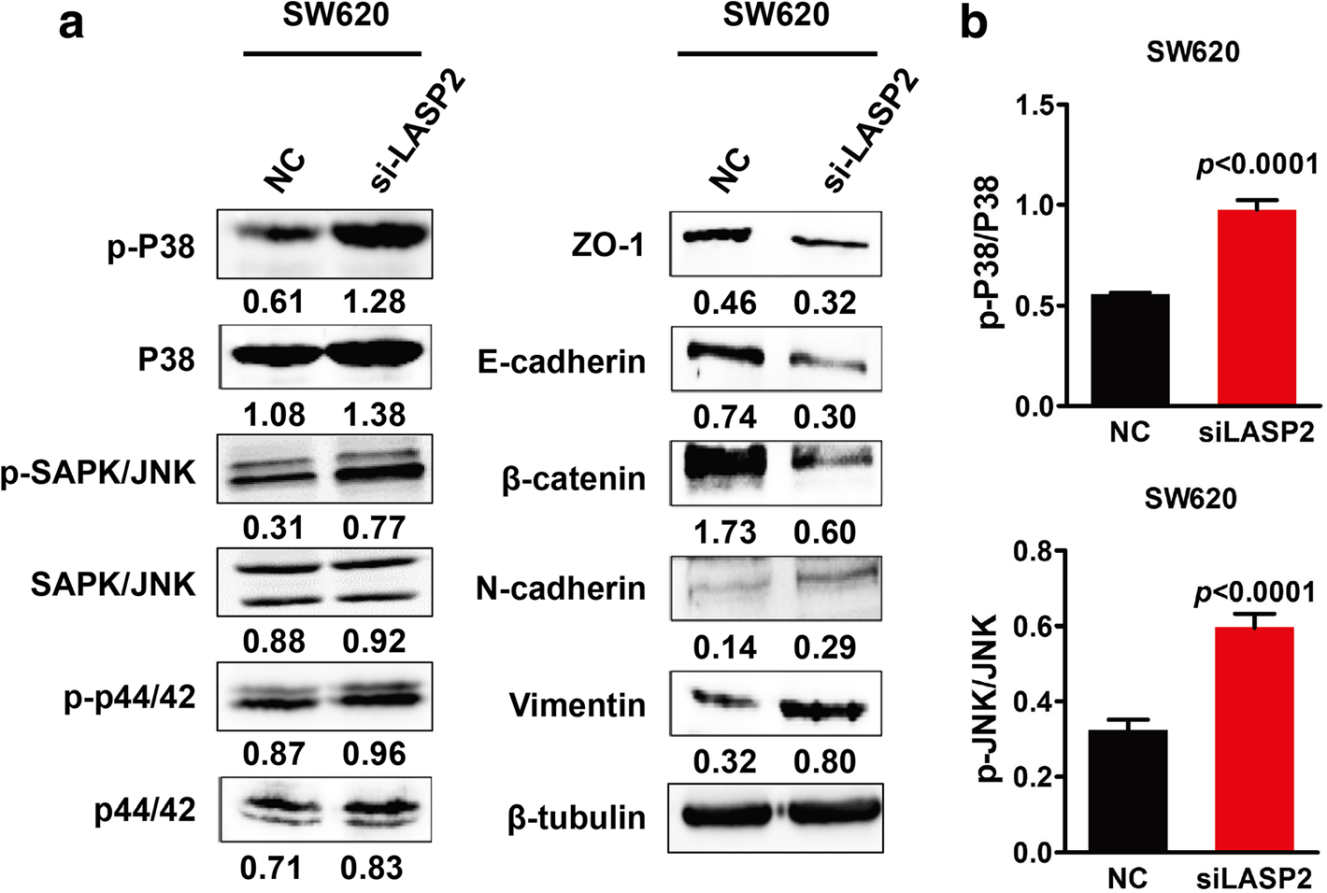
JNK/p38 MAPK inactivation is required for LASP2-medatied cell behaviors in CRC cells. **a** Marks of MAPK pathway and EMT were detected by western blot in LASP2 knockdown cell line SW620. Immunosignals of protein expression level were quantified by greyscale scanning software and relative protein abundance was normalized by β-tubulin. **b** Relative expression of phosphorylation of p38 and SAPK/JNK compared with original one. *Error bars* represent mean SD averaged from 3 independent experiments. (**) indicates *P* < 0.01

**Fig. 5:**
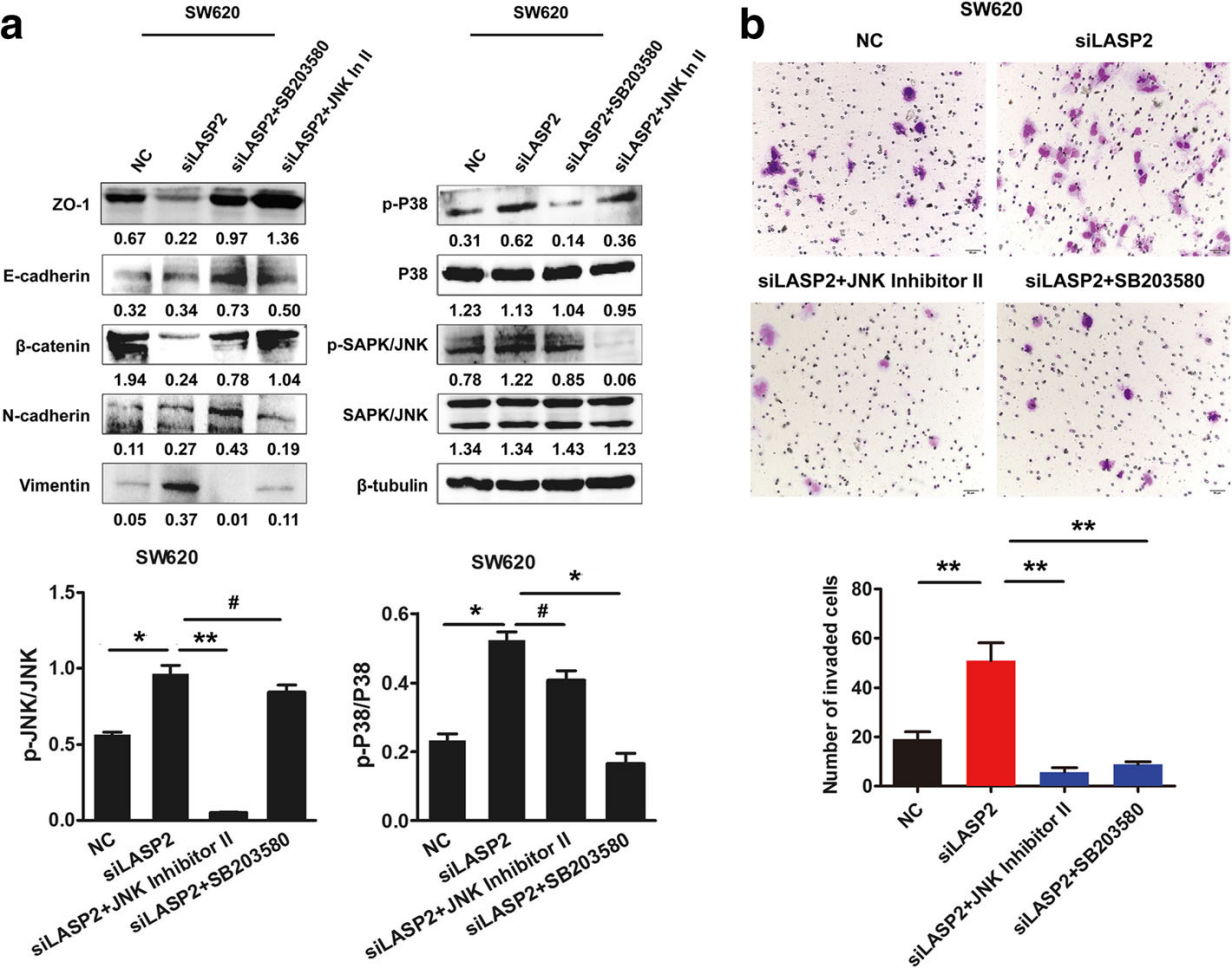
JNK/p38 MAPK inactivation is required for LASP2-medatied cell behaviors in CRC cells. **a** Western blot analysis of marks of MAPK pathway and EMT after treatment with JNK inhibitor JNK inhibitor II and p38 inhibitor SB203580 in LASP2 down-regulated CRC cell line SW620. Immunosignals of protein expression level were quantified by greyscale scanning software and relative protein abundance was normalized by β-tubulin. **b** Representative figures of transwell assay after treatment of JNK inhibitor JNK inhibitor II and p38 inhibitor SB203580. *Error bars* represent mean SD averaged from 3 independent experiments. (#) indicates not statistic significant while (*) indicate *P* < 0.05 and (**) *P* < 0.01

To discover the underlying mechanism of LASP2 suppressed CRC progression, western blot analysis was performed to detect the phosphorylation level of p38 and SAPK/JNK, two classical mark of the MAPK pathway. Results showed that knock down of LASP2 expression dramatically promoted the phosphorylation of p38 and SAPK/JNK in CRC cell (Fig. 4a and b), which could activate p38 and JNK signal pathway. Meanwhile, siRNA of LASP2 also decreased the expression of epithelial marker ZO-1, E-cadherin, β-catenin, and increased the expression of mesenchymal markers N-cadherin and Vimentin (Fig. 4a), subsequently acceleratting epithelial-mesenchymal transition (EMT). Furthermore, treatment of p38 inhibitor SB203580 and JNK inhibitor II profoundly prevented phosphorylation of p38 and SAPK/JNK and simultaneously the EMT (Fig. 5a), thus mitigated the aggressive phenotype brought by the depletion of LASP2 (Fig. 5b).

### LASP2 is negatively regulated by LASP1 in colorectal cancer cells and tissues

**Fig. 6:**
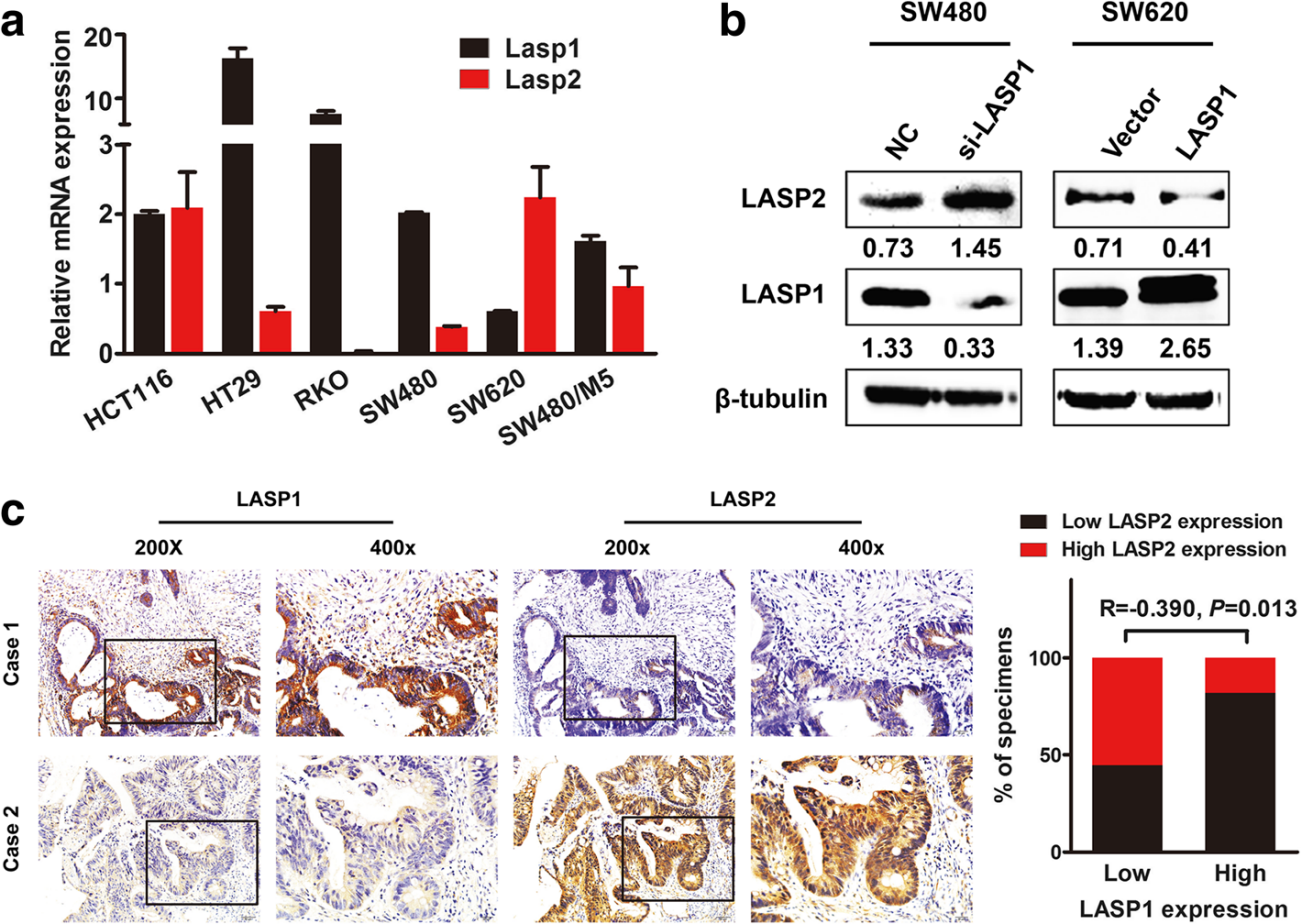
LASP2 is negatively regulated by LASP1 in colorectal cancer cells and tissues. **a** Relative mRNA expression of LASP1 and LASP2 in CRC cell lines tested by real time RT-PCR. *Error bars* represent mean SD averaged from 3 independent experiments. **b** LASP2 and LASP1 expression level was examined by western blot after transfected with LASP1 vector in SW620 and siRNA in SW480. Immunosignals of protein expression level were quantified by greyscale scanning software and relative protein abundance was normalized by β-tubulin. **c** Paraffin-embedded CRC samples were stained with anti-LASP1 or anti-LASP2 antibodies in the same position. Graphical illustration was shown in the right of LASP2 expression in the existence of LASP1 in 89 CRC samples

Considering the tumor suppressive function of LASP2 is not consistent with the promoted effect of LASP1, both of which belong to nebulin protein family, in cancer aggressiveness we expounded displayed previously [14], we evaluated the expression of LASP1 and LASP2 level in CRC cell lines and tissues. Results demonstrated that high expression level of LASP1 always accompanied with down-regulated level of LASP2 in both CRC cell lines and tissues (Fig. 6a and c). Both exogenously overexpressed and down-regulated of LASP1 expression in SW620 and SW480 resulted in reverse expression of LASP2 (Fig. 6b).

## Discussions

As a newly identified splice variant of nebulette most recently [21], LASP2 is rarely studied nowadays. Only several published papers of LASP2 were found on its structure as a binding scaffolding protein to actin filaments and Z-disc [9, 11, 22], let alone researches on its function. Since LASP2 shares a deep homology with LASP1 in the organization of protein with N-terminal LIM domain and nebulin repeats and C-terminal SH3 domain, LASP2 may be also functional in cell motility. Few studies have shed lights on the correlation between LASP2 and cell migration as well as spreading [10]. As we have a solid research basis on LASP1 in CRC cancer progression [12], the function of LASP2 was also studied in colorectal cancer. Our study draws a blank at present since no researchers have exerted themselves to the relationship between LASP2 and cancer, let alone its effects on clinical feature and prognosis. We are the first group that demonstrated the expression of LASP2 in CRC cell lines and colorectal cancer samples. Moreover, we further analyzed the correlation between LASP2 and prognosis of CRC patients, trying to evaluate LASP2 as a novel predictive factor in the diagnosis and progression of CRC.

Given that LASP2 could influence cell motility [10], we investigate its potential function in the proliferation and migration of CRC cells in vitro. Interestingly, we found an opposite effect of LASP2 compared to that of LASP1 despite they share a similar molecular composition. Their totally different function on CRC cell metastasis might result from their different distribution in focal adhesion [9]. We also tried to seek for molecular mechanism behind LASP2 induced cell motility. Since it has been reported that MAPK signaling pathway profoundly regulate cell proliferation and migration [23, 24], we screened all the marks of MAPK signaling pathway that might change during the knockdown of LASP2. As a result, the activation of JNK/p38 MAPK pathway was illustrate by the depletion of LASP2. Many epithelial tumors acquires an aggressive property to enter the surrounding stroma through epithelial-mesenchymal transition (EMT), thereby obtaining enhanced migratory and invasiveness capacity. Thus, the expression of EMT markers was also detected by western blot analysis in the present of pathway inhibitors to understand the mechanism behind the suppressive effect of CRC progression. We initiatively demonstrated that LASP2 could suppress colorectal cancer progression through JNK/p38 MAPK pathway meditated EMT.

Although LASP2 and LASP1 share high similarity in the structure [6], a few studies had discovered they had adverse distribution in some particular cellular position [9, 10]. Consistently, we detected an opposite distribution and function of LASP1 and LASP2 in CRC, which may be a part of the regulation networks in cancer progression. However, further works need to be done as to the interaction of LASP1 and LASP2, and we still have a long way to go to better understand the whole frame of cancer metastasis.

## Conclusion

Our research first demonstrated the expression of LASP2 in CRC cell lines and tissues, revealing a distinct function of LASP2 in CRC. We also further analyzed the correlation between LASP2 and clinical features as well as prognosis of CRC, which will add evidences to LASP2 as a novel biomarker in cancer staging and prediction of survival.

## Additional file

Additional file 1: Table S1. Relationship between LASP2 expression and the clinicopathological features of CRC patients. **Table S2.** Univariate and multivariate analyses of individual parameters for correlations with overall survival rate: Cox proportional hazards model. (DOCX 21 kb)

## Abbreviations

CRC: Colorectal cancer
EMT: Epithelial-mesenchymal transition
FBS: Fetal bovine serum
IHC: Immunohistochemistry
JNK: c-Jun N-terminal kinase
LASP1: LIM and SH3 Protein 1
LASP2: LIM and SH3 Protein 2
MAPK: Mitogen-activated protein kinases
OD: Absorbance value
siRNA: Small interfering RNA
