# Supplementary material for: LASP2 suppresses colorectal cancer progression through JNK/p38 MAPK pathway meditated epithelial-mesenchymal transition
Source: Cell Commun Signal. 2017 Jun 12;15:21. doi: 10.1186/s12964-017-0179-9 (PMC5469134; doi:10.1186/s12964-017-0179-9)
Supplement: Additional file 1: Table S1. — Relationship between LASP2 expression and the clinicopathological features of CRC patients. Table S2. Univariate and multivariate analyses of individual parameters for correlations with overall survival rate: Cox proportional hazards model. (DOCX 21 kb) [file 12964_2017_179_MOESM1_ESM.docx]

**Additional file 1**

**Table S1 Relationship between LASP2 expression and the clinicopathological features of CRC patients**

| **Features** | **n** | **LASP2 expression** | | **χ2** | **P-value** |
| --- | --- | --- | --- | --- | --- |
|  |  | **Low(%)** | **High(%)** |  |  |
| Gender |  |  |  |  |  |
| Male | 48 | 25(52.1) | 23(47.9) | 0.292 | 0.589 |
| Female | 41 | 19(46.3) | 22(53.7) |  |  |
| Age (years) |  |  |  |  |  |
| <50 | 13 | 9(69.2) | 4(30.8) | 1.549 | 0.213 |
| ≥50 | 76 | 35(46.1) | 41(53.9) |  |  |
| Tumor size (diameters) | |  |  |  |  |
| <5 cm | 52 | 23(44.2) | 29(55.8) | 1.357 | 0.244 |
| ≥5 cm | 37 | 21(56.8) | 16(43.2) |  |  |
| Differentiation |  |  |  |  |  |
| Poor | 13 | 11(84.6) | 2(15.4) | 7.536 | 0.023* |
| Moderate | 30 | 13(43.3) | 17(56.7) |  |  |
| High | 46 | 20(43.5) | 26(56.5) |  |  |
| T classification |  |  |  |  |  |
| T1+T2 | 9 | 5(55.6) | 4(44.4) | 0.001 | 0.972 |
| T3+T4 | 80 | 39(48.8) | 41(51.2) |  |  |
| N classification |  |  |  |  |  |
| N0 | 47 | 18(38.3) | 29(61.7) | 4.945 | 0.026* |
| N1+N2 | 42 | 26(61.9) | 16(38.1) |  |  |
| AJCC stage |  |  |  |  |  |
| I+II | 46 | 17(37.0) | 29(63.0) | 5.934 | 0.015* |
| III+IV | 43 | 27(62.8) | 16(37.2) |  |  |

* Statistically significant (P < 0.05).

**Table S2** Univariate and multivariate analyses of individual parameters for correlations with overall survival rate: Cox proportional hazards model

| Variables | Univariate | | |  | Multivariate | | |
| --- | --- | --- | --- | --- | --- | --- | --- |
|  | **OR** | **CI (95%)** | ***P* value** |  | **OR** | **CI (95%)** | ***P* value** |
| Gender | 0.412 | 0.181-0.935 | 0.034* |  | 0.419 | 0.184-0.956 | 0.039* |
| Age | 1.556 | 0.470-5.159 | 0.469 |  |  |  |  |
| Tumor size | 1.306 | 0.621-2.744 | 0.482 |  |  |  |  |
| Differentiation | 0.563 | 0.341-0.928 | 0.024* |  | 0.699 | 0.421-1.161 | 0.167 |
| T classification | 1.793 | 0.425-7.562 | 0.426 |  |  |  |  |
| N classification | 5.338 | 2.161-13.186 | 0.000* |  | 4.107 | 1.619-10.420 | 0.003* |
| LASP2 overexpression | 0.401 | 0.181-0.887 | 0.024* |  | 0.564 | 0.246-1.295 | 0.177 |

Abbreviations: OR, Odds ratio; CI, Confidence interval.

* Statistically significant (P < 0.05).
